# Case Report: Novel Dietary Supplementation Associated With Kidney Recovery and Reduction in Proteinuria in a Dialysis Dependent Patient Secondary to Steroid Resistant Minimal Change Disease

**DOI:** 10.3389/fped.2021.614948

**Published:** 2021-05-04

**Authors:** Rasheed A. Gbadegesin, Loren P. Herrera Hernandez, Patrick D. Brophy

**Affiliations:** ^1^Division of Nephrology, Department of Pediatrics, Duke University School of Medicine, Durham, NC, United States; ^2^Division of Anatomic Pathology, Mayo Clinic, Department of Pathology, Rochester, MN, United States; ^3^University of Rochester School of Medicine and Dentistry, Rochester, NY, United States

**Keywords:** minimal change disease, glycocalyx, antioxidants, kidney disease, nephrotic syndrome

## Abstract

Minimal change disease (MCD) is the most common cause of nephrotic syndrome worldwide. For decades, the foundation of the treatment has been corticosteroids. However, relapse rate is high and up to 40% of patients develop frequent relapsing/steroid dependent course and one third become steroid resistant. This requires treatment with repeated courses of corticosteroids, and second and third line immunomodulators increasing the incidence of drug related adverse effects. More recently, there have been reports of a very small subset of Nephrotic Syndrome (NS) patients who are initially steroid sensitive and later become secondarily steroid resistant. The disease course in this small subset is often protracted leading ultimately to end stage kidney disease requiring dialysis or kidney transplantation. Unfortunately, patients with this disease course do not do well post transplantation because 80% of them will develop disease recurrence that will ultimately lead to graft failure. Few approaches have been tried over many years to reduce the frequency of relapses, and steroid dependence and there is absolutely no therapeutic intervention for patients who develop secondary steroid resistance. Nonetheless, their therapeutic index is low, evidencing the need of a safer complementary treatment. Several hypotheses, including an oxidative stress-mediated mechanism, and immune dysregulation have been proposed to date to explain the underlying mechanism of Minimal Change Disease (MCD) but its specific etiology remains elusive. Here, we report a case of a 54-year-old man with steroid and cyclosporine resistant MCD. The patient rapidly progressed to end stage kidney disease requiring initiation of chronic dialysis. Intradialytic parenteral nutrition (IDPN), albumin infusion along with a proprietary dietary supplement, as part of the supportive therapy, led to kidney function recovery and complete remission of MCD without relapses.

## Introduction

Minimal change disease (MCD) is the most common cause of nephrotic syndrome (NS) in children and accounts for the 15–25% of cases of NS in adults (1–5). The specific etiology has remained elusive and a variety of different hypotheses have been generated to date (6). In general, MCD and by extension focal segmental glomerular sclerosis (FSGS) are thought to be related to damage of the podocytes. Both of these are considered podocytopathies and in the case of FSGS the etiology has been linked to genetic abnormalities of the podocyte apparatus and disruption of the slit diaphragm (1). There is suggestion that MCD may lead to FSGS in treatment resistant patients and at times it may be difficult to adequately obtain a kidney biopsy sample that reflects the progression of MCD toward FSGS (due to potential biopsy sampling error) (1). Recent evidence from genetic association studies suggests that genetic variation in the adaptive immune response coupled with environmental factors may be very important risk factors for development of MCD (7–10). The animal model that most closely resembles MCD is the puromycin aminonucleoside nephrosis (PAN) in rats, which leads to the production of reactive oxygen species and direct DNA damage. This subsequently leads to the pathologic characteristics of MCD (i.e., alteration of the podocyte actin cytoskeleton, foot process effacement, and detachment from the glomerular basement membrane). These pathologic changes result in proteinuria. Interestingly, Angiopoietin-like 4 (Angptl4) is a secreted glycoprotein that is highly upregulated in the glomeruli of several models of podocyte injury in rats, including PAN, and in the human disease (1). This upregulation is specific to models of steroid-sensitive NS. Recently, it has been demonstrated that syndecan-1 level, a biomarker of endothelial glycocalyx damage, is increased in nephrotic patients with near-normal kidney function and it plays an important role in endothelial dysfunction (11). Angiopoietin-2 (ANGPT2) is an endothelial growth factor that promotes cell derangement (12). Indeed, the linkage between endothelial glycocalyx damage, proteinuria and systemic vascular dysfunction is becoming clearer (13). Therefore, loss of the endothelial surface layer appears to be a link between albuminuric kidney disease and systemic vascular dysfunction, providing a potential therapeutic target for proteinuric kidney disease. Besides glycoprotein dysregulation, immunologic hypotheses exist involving regulatory T-Cell and B cell immune dysregulation that have also been implicated in the pathophysiology of MCD (14).

The cornerstone of the treatment has been corticosteroids (e.g., prednisone). Although MCD in children is usually sensitive to corticosteroids, resulting in remission within few weeks, adults have delayed response to the treatment, sometimes requiring 24 weeks or more of treatment to enter remission. Moreover, relapse rate in adults may be up to 70%, with one third becoming frequent relapsing (15). This requires treatment with repeated courses of corticosteroids, increasing the incidence of drug related adverse effects, including weight gain, diabetes mellitus, infection, osteoporosis, vascular necrosis and gastrointestinal bleeding (16, 17).

Few approaches have been tried over many years to reduce the frequency of relapses and/or permanently cure MCD, such as alkylating agents (e.g., cyclophosphamide) and calcineurin inhibitors (e.g., cyclosporine, tacrolimus). In particular, cyclosporine is frequently used in patients that are either frequent relapsing, steroid dependent or resistant. However, its therapeutic index is low. Therefore, patients undergoing long-term therapy with cyclosporine are susceptible to drug-related toxicity; including diabetes, hypertension, dermatologic complications, gastrointestinal, hepatic, neurological complications, hyperlipidemia, electrolyte abnormalities, neoplasia, and acute or chronic nephrotoxicity. In many instances, it is difficult to distinguish whether the chronic injuries are caused by cyclosporine or by the progression of the disease (18). Emma and colleagues present an excellent review of protocolized approaches to treatment in adults (1).

Acute kidney injury (AKI) can occur and is not uncommon in adults with MCD, affecting up to 25% of patients, while progressive CKD in not common in adults with MCD; its occurrence is usually associated with underlying FSGS. Sometimes, AKI is severe enough to require dialysis. However, kidney function is usually recoverable within 14–21 days, although patients with AKI may progress to CKD due to residual kidney damage (19).

In certain circumstances, IDPN and albumin infusion have been used as supportive treatment for MCD and AKI. Nonetheless, their use remains controversial due to the risks associated with the treatment, their temporary effect, and the high cost. Interestingly, it is estimated that about 40% of patients with MCD may undergo spontaneous remission with supportive care (20, 21). However, that usually happens only after an unacceptable time of uncontrolled nephrotic syndrome, increasing the incidence of infection, thrombosis, malnutrition, and kidney injury.

Here, we report a case of a previously healthy 54-year-old man with steroid and cyclosporine resistant MCD and nephrotic range proteinuria. The patient did not respond to standard therapy with diuretics, corticosteroids, and Calcineurin inhibitors (CNI) but rapidly progressed to end stage kidney disease requiring initiation of chronic dialysis and transplant work-up and listing. IDPN, albumin infusion and a proprietary dietary supplement [the supplement consists of a patent pending proprietary formula composed of 11 food grade supplements/ingredients in the proprietary formula that optimize the vascular system (see Appendix 1)] along with restoration of colloidal stability initiated by the patient himself, as part of the supportive therapy led to kidney function recovery and complete remission of MCD without relapses.

## Case Report

**Figure 1:**
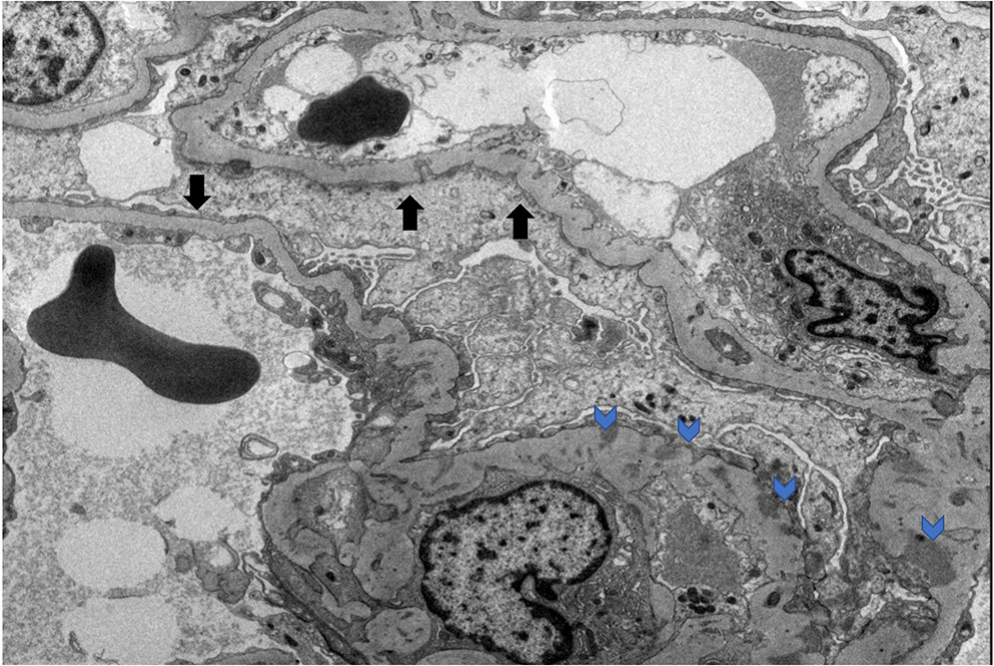
MCD electron microscopy. Black arrows denote podocyte effacement. Blue arrows denote podocyte subendothelial deposits.

On October 2014, a 54-year-old male with no significant past medical history presented to the hospital with bilateral lower extremity edema and chest pain. A clinical, historical and imaging assessment revealed no evidence of a secondary cause of his clinical presentation. On admission his blood pressure was 157/93 mmHg. Laboratory tests revealed hypoalbuminemia (1.1 g/dl), elevated D-dimer (5,250 ng/ml), creatinine (1.4 mg/dl), BUN (19 mg/dl), low calcium (7.7 mg/dl), and proteinuria with random urine protein/creatinine ratio 5,139 mg/g. An ultrasound and a CT of the abdomen with IV contrast ruled out DVT in both extremities and any compressing mass or thrombus in the inferior vena cava, respectively. The patient was treated with furosemide 20 mg p.o. daily without improvement. Kidney biopsy was performed, revealing global effacement of the epithelial cell foot processes, unremarkable glomeruli on light microscopy, focal loss of fenestration of the endothelial cells and signs of injury with mild ectasia and slight vacuolization of the tubules consistent with minimal change disease (Figure 1).

The MCD was considered idiopathic and initially treated with mycophenolate and prednisone (80 mg p.o. daily) for 8 weeks without any improvement. In December 2014, the patient continued to have problems with anasarca and volume overload; he was given outpatient diuretics including furosemide, torsemide, and metolazone, and he initially lost weight. In January 2015, his 24-h urine protein went up to 12 g/day and he was started on cyclosporine 50 mg p.o. twice daily. After gaining 50 pounds through fluid retention, he was admitted to the hospital and treated with IV furosemide drip 20 mg/h along with IV albumin, metolazone, prednisone (40 mg) and cyclosporine (75 mg twice daily). The patient was also put on a low salt diet with fluid restriction and he lost 50 pounds of fluids during the 13 days of hospitalization. His serum albumin went up to 2.7 g/dl and creatinine improved to 1.0 mg/dl. However, proteinuria was not resolved. He was discharged with the following medications: cyclosporine (100 mg twice daily), prednisone (40 mg daily), and furosemide (80 mg daily).

**Figure 2:**
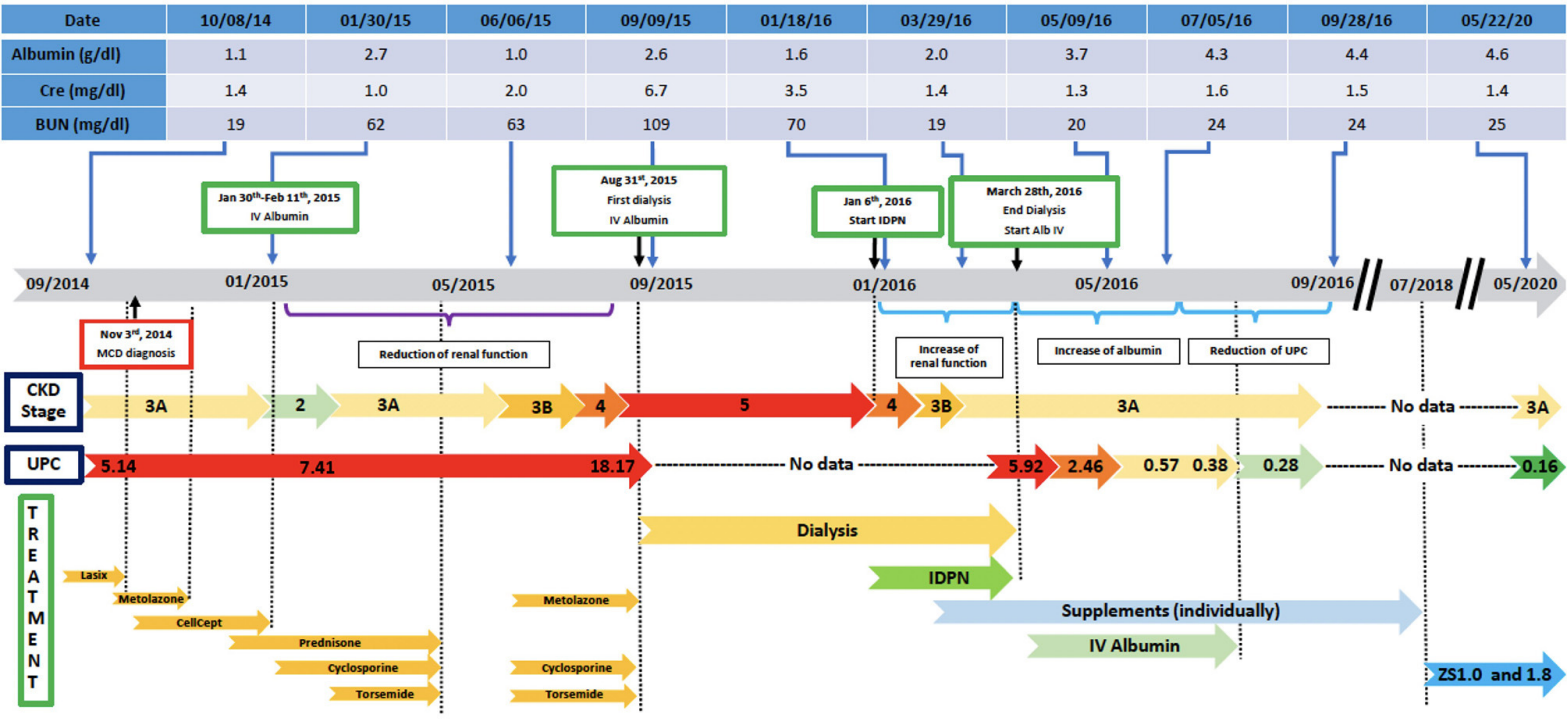
Disease course and treatment.

**Figure 3:**
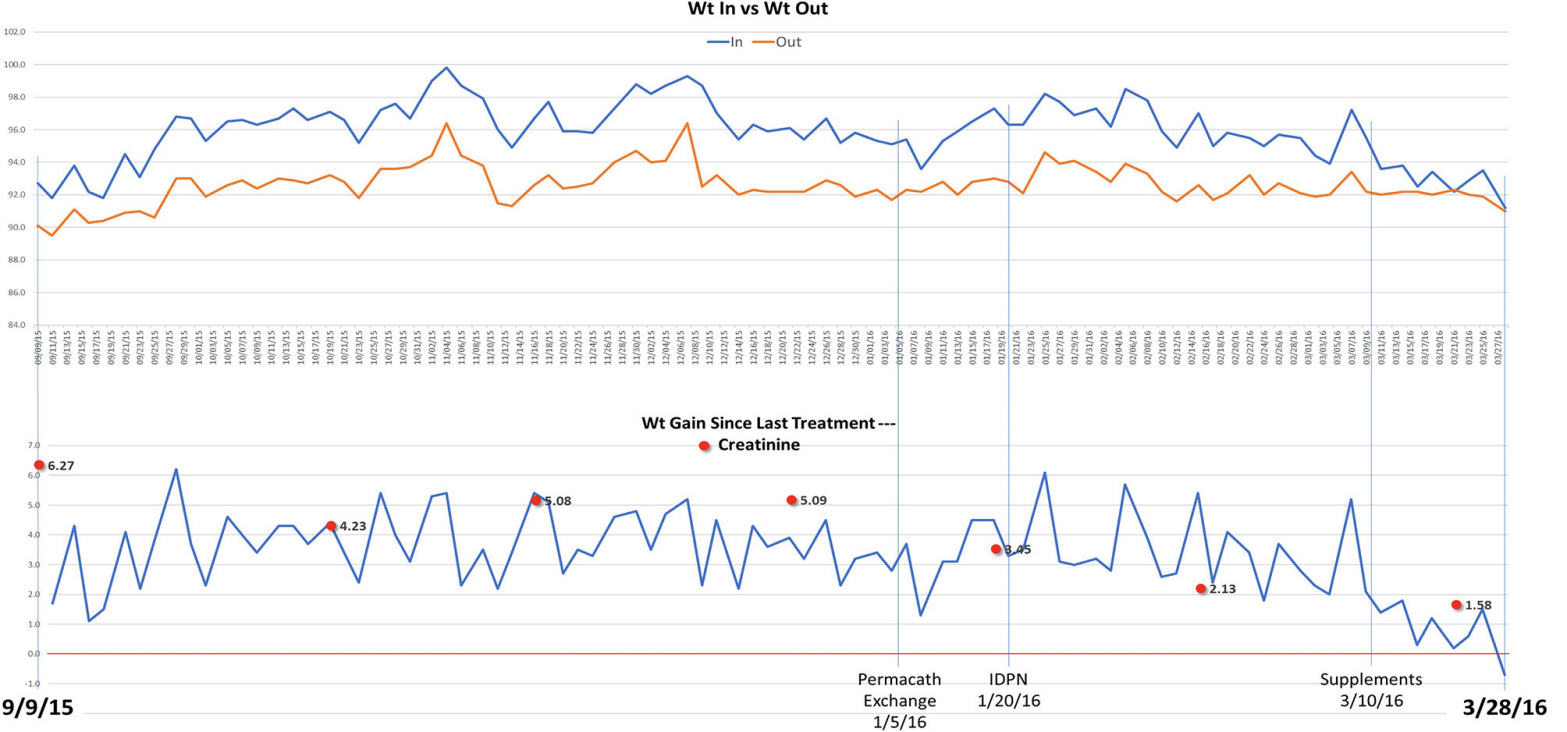
Dialysis timeline and weights.

In the following months, prednisone was tapered off and eventually discontinued while cyclosporine was increased to 125 mg twice daily. On a follow up visit in March 2015, laboratory tests revealed increased BUN (51 mg/dl) and creatinine (1.5 mg/dl), and low chloride (91 meq/l), potassium (3.1 meq/l), calcium (7.5 mg/dl), and albumin (1.5 g/dl). Subsequently, the patient lost his health insurance and discontinued cyclosporine. In June 2015, his BUN was 63 mg/dl and creatinine 2.0 mg/dl and he was treated with cyclosporine (200 mg twice daily), metolazone (5 mg daily) and torsemide (100 mg twice daily) for 30 days. The patient rapidly progressed to kidney failure (BUN 109 mg/dl and creatinine 6.27 mg/dl) and, in September, initiated hemodialysis thrice weekly given the patient's excess fluid overload and inability to control his metabolic status. The patient's daily ultrafiltration rate was up to 5 l/day. The patient continued to require significant fluid and electrolyte control and after 3 months on dialysis, was evaluated and listed for kidney transplantation and was placed on the waiting list. When the patient was placed on chronic dialysis, the patient (who has an extensive scientific engineering background) began to review the literature on MCD and proteinuria. Over time he developed a more comprehensive understanding of the pathophysiology of MCD and proteinuria. He developed a hypothesis and with a colleague who had access to a nutritional supplement process facility, began to evaluate possible treatment approaches. Together they developed and continue to produce this proprietary nutritional supplement (in a regulated sterile production facility). It was at this point that he, on his own began a supplement regimen to promote regrowth of the glycocalyx and prevent its degradation (Appendix 1) in the presence of a balanced colloid and electrolyte environment. Due to poor nutrition and at the request of the patient, at the beginning of January 2016, he initiated standard IDPN therapy together with hemodialysis. He responded to the improved nutritional delivery and intermittent hemodialysis and his kidney indices improved after 2 weeks of treatment (BUN 70 mg/dl and creatinine 3.45 mg/dl) and at the end of April BUN was 19 mg/dl and creatinine 1.4 mg/dl. Therefore, hemodialysis was discontinued. However, proteinuria was still present (random urine protein/creatinine ratio was 5,919 mg/g) and serum albumin was low (2.0 g/dl). The patient received supportive care consisting of IV albumin thrice weekly and His serum albumin increased up to 5.0 g/dl on July 2016 and IV albumin was discontinued. Proteinuria progressively decreased from June (random urine protein/creatinine ratio 2,458 mg/g) and almost completely resolved at his last follow up visit on September 2016 (random urine protein/creatinine ratio 280 mg/g). The patient continued to administer himself supplements and, as of today, he has had no further relapses. On May 2020, his urine protein/creatinine ratio was 159 mg/g, and his kidney function was stable (BUN 25 mg/dl and creatinine 1.41 mg/dl). Figure 2: Timeline for treatment and response. Figure 3 Chronic dialysis course.

## Discussion

Here, we present the case of a previously health 54 yr old male who developed idiopathic biopsy proven MCD. Despite standardized treatment regimen he went on to develop progressive CKD and qualified for ESKD per KDIGO definition and was listed for kidney transplantation. While he met the KDIGO criteria, no biopsy was conducted and it is possible he had prolonged AKI which recovered with no ongoing indications of CKD, which in such a case would be unusual. During the course of his work up, he was assessed for possible infection related causes for initiation of his MCD. No etiologic agents (EBV, Hepatitis, HIV) were identified. He required IDPN and initiated a proprietary dietary supplement regimen based on his blood biomarkers. The goal of the proprietary dietary supplement regimen was to restore the glycocalyx along with the electrolyte balance to not only come off of chronic dialysis, but also to resolve his proteinuria and MCD. He has remained stable off all medication except for his daily supplement regimen. He has had no further relapses and has had resolution of his proteinuria. Importantly, the patient has tolerated the supplements without any notable deleterious side effects. Several components of the supplement included in the regimen are potent antioxidants. This case example furthers the association of antioxidants and their role in the treatment and prevention of proteinuric kidney disease and other vascular inflammatory states.

Mechanisms underlying the relation between kidney disease and systemic endothelial cell dysfunction remain incompletely understood, but a defect in the endothelial surface layer, which is common to all blood vessels, has been suggested as a mechanistic link between widespread vascular dysfunction and albuminuric kidney diseases leading to CKD and ESKD (22). The data for the value of antioxidant-based therapy in treating kidney and endovascular diseases are becoming clearer. Indeed, oxidative stress is regarded as a key mediator of endothelial dysfunction in kidney disease and contributes to development and progression of CKD (23). A Cochrane meta-analysis reviewed 10 randomized controlled (*n* = 1,979) trials investigating antioxidant use (vitamin E, co-enzyme Q, N-acetylcysteine, and human recombinant superoxide dismutase) in CKD (24). Data were pooled using the random effects model and expressed as either risk ratios (RR) or mean difference (MD) with 95% confidence intervals (CI) (24). The authors found that “compared with placebo, antioxidant therapy showed no clear overall effect on cardiovascular mortality (RR 0.95, 95% CI 0.70–1.27; *P* = 0.71); all-cause mortality (RR 0.93, 95% CI 0.76–1.14; *P* = 0.48); cardiovascular disease (RR 0.78, 95% CI 0.52–1.18; *P* = 0.24); coronary heart disease (RR 0.71, 95% CI 0.42–1.23; *P* = 0.22); cerebrovascular disease (RR 0.91, 95% CI 0.63–1.32; *P* = 0.63); or peripheral vascular disease (RR 0.54, 95% CI 0.26–1.12; *P* = 0.10)” (24). The authors concluded that while there was a potential benefit for the use of antioxidant supplementation in the prevention of progression from CKD to ESKD the studies lacked power to establish any definitive conclusions (24). It may be that a combinational therapeutic approach would give better results as antioxidants would prevent further ongoing damage in the face of restoration of the GCX and colloidal stability at the same time. Individuals with genetic susceptibility to oxidative stressors may be benefited from enhancing the anti-oxidant milieu present in their bodies, especially those anti-oxidants that target specific pathways. Increased oxidative stress is a major contributor to CKD progression and in patients undergoing chronic dialysis (25). In humans, a common deletion variant of the glutathione-S-transferase μ-1 (GSTM1) gene results in decreased GSTM1 enzymatic activity and is associated with elevated levels of oxidative stress factors (26). GSTM1 are antioxidant enzymes that are regulated by nuclear factor erythroid 2-related factor 2 (NRF2). In both the African American Study of Kidney Disease (AASK) trial and the Atherosclerosis Risk in Communities (ARIC) study participants GSTM1 null mutations were associated with enhanced CKD progression (25, 27). These findings have also been demonstrated in mouse Gstm1 knockout animals (28). Recently, glucoraphanin, a precursor of sulforaphane (found in broccoli and other like vegetables- brussels sprouts, cabbage) have been shown to have protective effects against oxidative damage through activation of NRF2 (27–29). Interestingly, the use of purified broccoli extract in Gstm1 knockout mice can reduce renal disease, a finding that correlates to a slowing of progression in GSTM1 null patients in the ARIC study that had high daily intake of cruciferous vegetables (27, 28). While genetic underpinnings have revealed the potential impact of dietary supplements on possible progression of renal disease, it remains unclear as to their effects on patients with other genotypes. There are now multiple studies examining the effects of purified broccoli powder as a supplement for mitigation and prevention of inflammatory states in a variety of diseases^1^. We speculate that the interplay between a triggered inflammatory/immune response leads to antioxidant depletion with subsequent glycocalyx defects and a loss of colloidal balance resulting in propagation of the disease state.

Damage to the endothelial glycocalyx in response to factors in albuminuric plasma provides a novel concept in understanding endothelial dysfunction in disease states. In fact, albumin (which is dramatically lost in NS) is the most abundant and therefore main antioxidant (as it has a thiol group that can bind to ROS). Ghiggeri's group (30) has demonstrated the presence of massive oxidation of albumin associated with FSGS, implicating oxidative mechanisms. Therefore, a lack of albumin may exacerbate the antioxidant imbalance.

We present a case report of a proprietary dietary supplement (consisting of a patent pending proprietary formula composed of 11 food grade supplements/ingredients in the proprietary formula that optimize the vascular system) regimen associated with recovery from ESKD and MCD. Our hypothesis builds upon current evidence that the albuminuric state itself might lead to systemic alterations in endothelial cell function, likely involving a combination of mechanisms. We are now developing animal modeling, to better understand the pathways underlying the effects of albuminuric plasma on the endothelial glycocalyx, assessing whether the endothelial glycocalyx is damaged in blood vessels *in situ* in patients with kidney disease, and examining whether the endothelial glycocalyx can be restored (and endothelial cell function improved) by manipulating these pathways. Our long-term goal is to manipulate endothelial glycocalyx regulation to develop an entirely new class of treatments for vascular protection in difficult to treat nephrotic syndrome and other chronic kidney diseases.

## Data Availability Statement

The original contributions presented in the study are included in the article/Supplementary Material, further inquiries can be directed to the corresponding author/s.

## Ethics Statement

Written informed consent was obtained from the individual(s) for the publication of any potentially identifiable images or data included in this article.

## Author Contributions

All authors listed have made a substantial, direct, and intellectual contribution to the work and approved it for publication.

## Conflict of Interest

PB consults for Zeta Biolongevity, Inc. and receives royalties from UpToDate.

The remaining authors declare that the research was conducted in the absence of any commercial or financial relationships that could be construed as a potential conflict of interest.

## Footnotes

^1^https://www.clinicaltrials.gov/ct2/results?term=SFN+broccoli+extract
