# Supplementary material for: Case Report: Novel Dietary Supplementation Associated With Kidney Recovery and Reduction in Proteinuria in a Dialysis Dependent Patient Secondary to Steroid Resistant Minimal Change Disease
Source: Front Pediatr. 2021 May 4;9:614948. doi: 10.3389/fped.2021.614948 (PMC8129002; doi:10.3389/fped.2021.614948)
Supplement: Supplementary file 1 [file Table_1.DOCX]

Appendix 1: Specific blend proportions of constituents are proprietary

Components of Proprietary supplement:

Electrolytes

Olive Leaf extract

Methyl Sulfonyl Methane

Chondroitin Sulfate

Glucosamine Sulfate

NAG

Hyaluronic Acid

N-Acetyl Cysteine

Lactoferrin

Tocotrienols

Nattokinase
